# Supplementary material for: Splice-Junction-Based Mapping of Alternative Isoforms in the Human Proteome
Source: Cell Rep. Author manuscript; Available in PMC 2020 Jan 15. (PMC6961840; doi:10.1016/j.celrep.2019.11.026)
Supplement: 2 [file NIHMS1546469-supplement-2.zip › DF1/PXD006675/RightAtrium/RightAtrium_43_RILPL1_ASSDAAESALEK.pdf]

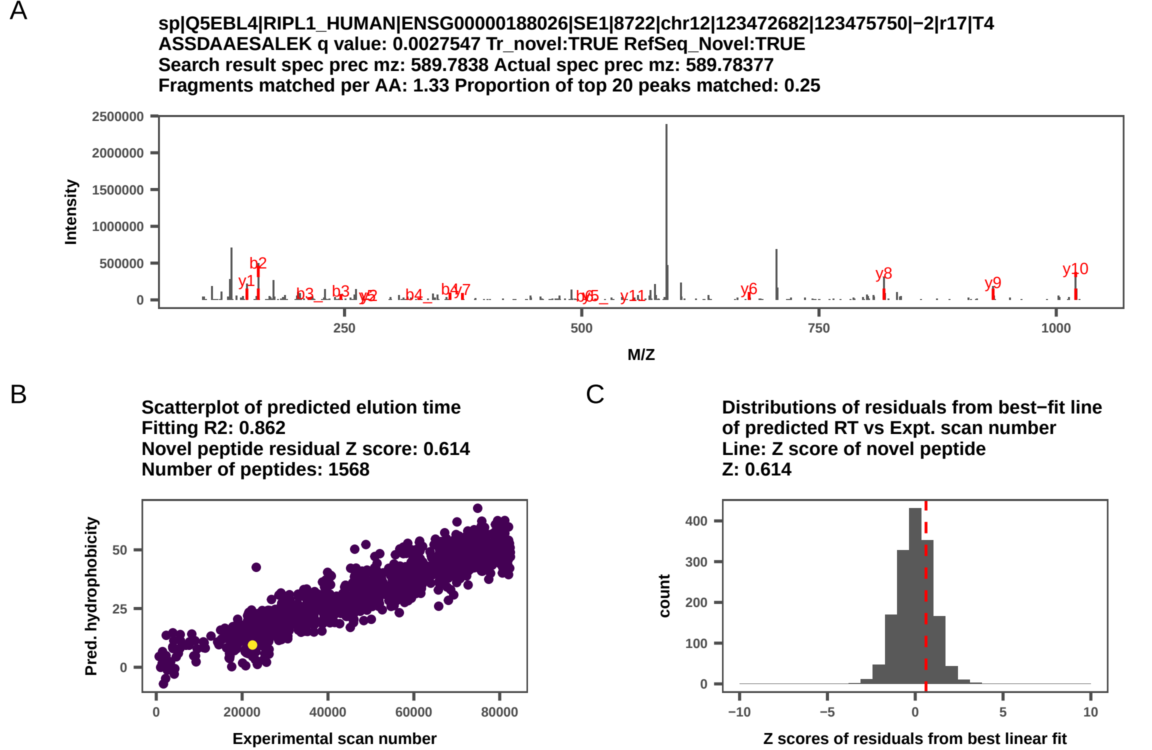
A
sp|Q5EBL4|RIPL1_HUMAN|ENSG00000188026|SE1|8722|chr12|123472682|123475750|−2|r17|T4
ASSDAAESALEK q value: 0.0027547 Tr_novel:TRUE RefSeq_Novel:TRUE
Search result spec prec mz: 589.7838 Actual spec prec mz: 589.78377
Fragments matched per AA: 1.33 Proportion of top 20 peaks matched: 0.25
Intensity
2500000
2000000
1500000
1000000
500000
0
b2
y1
b3_
b3
y2
b4_
y8
y6
y9
y10
y11
250
500
750
1000
M/Z
B
Scatterplot of predicted elution time
Fitting R2: 0.862
Novel peptide residual Z score: 0.614
Number of peptides: 1568
Pred. hydrophobicity
50
25
0
0
20000
40000
60000
80000
Experimental scan number
C
Distributions of residuals from best−fit line
of predicted RT vs Expt. scan number
Line: Z score of novel peptide
Z: 0.614
count
400
300
200
100
0
−10
−5
0
5
10
Z scores of residuals from best linear fit
